# Assessing the Spectrum of Internet Use in a Healthy Sample: Altered Psychological States and Intact Brain Responses to an Equiprobable Go/NoGo Task

**DOI:** 10.3390/bs15050579

**Published:** 2025-04-25

**Authors:** Dovile Simkute, Povilas Tarailis, Evaldas Pipinis, Inga Griskova-Bulanova

**Affiliations:** 1 Life Sciences Center, Institute of Biosciences, Vilnius University, LT-10257 Vilnius, Lithuania; 2 Translational Health Research Institute, Faculty of Medicine, Vilnius University, LT-08406 Vilnius, Lithuania

**Keywords:** behavioral addiction, problematic internet use, PIU, electroencephalography, EEG, equiprobable Go/NoGo paradigm, event-related potentials, ERPs

## Abstract

Problematic internet use (PIU) is linked to psychological distress and cognitive alterations, yet its early pre-clinical effects remain unclear. This study explored the psychological, behavioral, and neurophysiological correlates of PIU in a healthy, non-clinical population, focusing on response inhibition and execution within internet use patterns. A total of 133 participants (74 females, aged 18–35) were assessed using PIUQ-9 and DPIU questionnaires, along with measures of anxiety, depression, and obsessive–compulsive symptoms. An auditory equiprobable Go/NoGo task was used and event-related potentials (ERPs; N1/N2/P2/P3) were analyzed in relation to PIU severity and different online activities engagement. Additionally, behavioral, psychological, and neurophysiological profiles of individuals with high and low PIU levels were compared. PIU severity correlated with anxiety, depression, and obsessive–compulsive symptoms, while Go/NoGo task accuracy was unaffected. N1 amplitudes negatively correlated with PIU severity and gaming engagement, suggesting altered early sensory processing. NoGo-P3 latency positively correlated with information search engagement, indicating delayed inhibitory processing in frequent online searchers. High and low PIU groups differed in psychological measures but not in ERP or behavioral measures. Our findings confirm psychological distress in PIU alongside subtle neurophysiological alterations, suggesting that ERP measures in the equiprobable Go/NoGo task may not be highly sensitive PIU risk biomarkers in non-clinical populations.

## 1. Introduction

The number of internet users reached 5.35 billion in 2023 (Petrosyan & Statista, 2024) and is projected to rise to 7.3 billion by 2029 (Statista Research Department, n.d.). While internet integration offers numerous benefits, concerns regarding problematic internet use (PIU) are increasing. Recognized as a public health issue (World Health Organization, 2015), PIU encompasses excessive and dysfunctional online behaviors, often linked to impulse control deficits, cognitive dysfunction (Ioannidis et al., 2019), and psychiatric comorbidities, such as anxiety, ADHD, depression, and insomnia (Feher et al., 2023; Fineberg et al., 2022; Kuss & Lopez-Fernandez, 2016).

Despite this continuum-based progression, most studies have focused on individuals with established clinical PIU (e.g., Internet Addiction Disorder or Internet Gaming Disorder), overlooking gradual neural and cognitive changes that might occur in non-clinical populations. PIU does not emerge abruptly—it develops gradually along a continuum, ranging from casual but excessive use to severe, compulsive engagement (Anderson et al., 2017). A sensitive biomarker—providing an objective and quantifiable measure of underlying neural dysfunction that may precede the overt behavioral symptoms of PIU—could reflect this progressive nature by showing gradual alterations with increasing internet engagement, rather than only differentiating clinical cases from controls. However, such progressive neurophysiological markers—capable of detecting subtle, pre-clinical changes in brain activity, such as alterations in attention, impulse control, or error monitoring that do not yet manifest in self-report measures or clinical diagnosis—remain underexplored.

One promising approach for distinguishing problematic from non-problematic internet use is through the lens of response inhibition. Response inhibition is defined as the capacity to suppress impulsive reactions incompatible with long-term goals and to facilitate goal-directed behavior by resisting both internal and external distractions (Brand et al., 2019; Hofmann et al., 2012). Impulsivity and deficits in inhibitory control are central features implicated in the development and maintenance of numerous psychiatric conditions, including a range of addictive behaviors, such as smoking, alcohol and drug use, and gambling, with consistent findings at both behavioral and neurobiological levels (Beppi et al., 2020; Brevers & Noël, 2013; Fillmore & Rush, 2002; Jentsch & Pennington, 2014; Spinella, 2002; Winstanley et al., 2006). Considering PIU, studies report altered P3 amplitudes during Go/NoGo or stop-signal tasks in individuals with PIU or internet related problematic behaviors (Dong et al., 2010; Park et al., 2016, 2017a; Yu et al., 2024), indicating impairments in attentional resources and diminished inhibitory processing efficiency (J. S. Fogarty et al., 2020; Randall & Smith, 2011). Additionally, reduced Error-Related Negativity (ERN) amplitudes in PIU (Park et al., 2020; Zhou et al., 2013) reflect response monitoring deficit and maladaptive performance adjustments, potentially contributing to excessive internet platforms engagement. The N2 component, considered to reflect conflict detection and early inhibitory processes (Donkers & van Boxtel, 2004; Enriquez-Geppert et al., 2010), has also been found either attenuated or delayed in individuals with PIU behaviors, further supporting the hypothesis of inhibitory control deficits (Yu et al., 2024; Zhou et al., 2010). However, while alterations in response inhibition-related neural markers have been observed in clinical PIU cases, findings are inconsistent (for example, results by Dong et al., 2010; Kim et al., 2017 versus Littel et al., 2012) and little is known about how these markers evolve across different levels of internet use.

This study aims to bridge this gap by investigating how cognitive control mechanisms vary with different intensities of internet engagement in a healthy, non-clinical population. More specifically, does the severity of internet use relate to sensory and inhibitory processing at the neurophysiological level, and do these alterations emerge gradually along the PIU continuum? Using the equiprobable Go/NoGo task, a well-established method for assessing sensory processing and response inhibition (Barry & De Blasio, 2013), we examine event-related potentials (ERPs), behavioral performance, and depression and anxiety symptoms across individuals with varying levels of internet use. By identifying gradual neurophysiological changes rather than categorical differences, this study seeks to establish early biomarkers of problematic internet engagement, paving the way for interventions before significant impairment occurs.

## 2. Materials and Methods

### 2.1. Participants

A total of 161 subjects participated in the study (males: 71 and females: 90). Data from 28 subjects were excluded due to technical issues (n = 5), poor recording quality (n = 17), or inadequate task performance (<40 correct epochs; n = 6), resulting in a final sample of 133 participants (M:F = 59:74) and a 17.39% data loss. Participants were aged 18–35 years (mean: 24.19 ± 4.27 years).

Inclusion criteria required good general health, normal or corrected-to-normal vision, and normal hearing. Female participants were required to have a regular menstrual cycle for at least three months prior to the experiment and could not use hormonal contraception. As hormonal fluctuations across different menstrual phases have been shown to significantly influence cognitive processes and electrophysiological correlates of executive functioning (Griskova-Bulanova et al., 2016), all female participants were tested during the early follicular phase, specifically within the first days of menstruation, to reduce the potential confounding effects and improve the reliability and consistency of neurophysiological data. Exclusion criteria included history or diagnosis of psychiatric, neurological, or endocrinological disorders, use of psychotropic or psychoactive substances (except nicotine), and self-reported or clinically confirmed addictions.

Participants were recruited via student community advertisements, social media, and other platforms. They were instructed to ensure a good night’s sleep and to avoid caffeine, energy drinks, and nicotine for at least two hours before the experiment. The study was approved by the Vilnius Regional Biomedical Research Ethics Committee (Nr. 2019/10-1159-649), and all subjects provided written informed consent.

### 2.2. The Equiprobable Auditory Go/NoGo Task

The auditory equiprobable Go/NoGo task was selected for its well-established application across diverse populations and its extensively studied neural mechanisms (Barry & De Blasio, 2013; J. Fogarty, 2020; J. S. Fogarty et al., 2018). The equiprobable Go/NoGo variant, characterized by equal stimulus probabilities, has emerged as particularly advantageous for delineating sensory and cognitive processes without probability-induced response biases, thus offering enhanced clarity on cognitive demands (Barry & De Blasio, 2013; J. S. Fogarty et al., 2018, 2020). Event-related potential (ERP) components—N1 (early sensory processing), P2/N2 (response execution), and P3 (response termination)—were assessed.

The paradigm consisted of 150 auditory stimuli (50% Go and 50% NoGo), with 75 Go and 75 NoGo trials. Following Barry et al. (2007) and our previous studies (Griskova-Bulanova et al., 2016; Melynyte et al., 2017), two binaural tones (1000 Hz and 1500 Hz, 60 dBA, 50 ms duration, and 5 ms rise/fall time) were presented in a random order with a fixed stimulus onset asynchrony (SOA) of 1100 ms. The assignment of tone frequency to Go or NoGo conditions was counterbalanced across participants.

Participants were instructed to respond as quickly and accurately as possible by pressing a key for Go stimuli and withholding responses for NoGo stimuli, while maintaining fixation on a white cross (+) on a black screen. A practice block of 5 trials preceded the task.

Responses were categorized as follows: correct Go—key pressed within 1000 ms of Go stimulus onset; correct NoGo—no response to a NoGo stimulus; incorrect Go (Omission Errors)—no response within 1000 ms of Go stimulus onset; and incorrect NoGo (Commission Errors)—key pressed during a NoGo stimulus.

### 2.3. EEG Recording and Processing

Participants were comfortably seated in an upright position within a dimly lit, electrically shielded, and sound-attenuated chamber. EEG data were recorded using a 64-channel WaveGuard EEG cap with silver/silver chloride (Ag/AgCl) electrodes, following the International 10-10 System, and acquired using ANT Neuro (The Netherlands) equipment. Mastoid electrodes (M1 and M2) served as reference, and the ground electrode was positioned near Fz. Electrode impedances were maintained below 20 kΩ, and the data was sampled at 2048 Hz.

An electro-oculogram (EOG) was recorded to monitor ocular artifacts, with vertical EOG (VEOG) electrodes placed above and below the right eye and horizontal EOG (HEOG) electrodes positioned at the outer canthi of both eyes.

EEG data were processed offline using EEGLAB (Delorme & Makeig, 2004) within MATLAB (version R2020a, The MathWorks, Natick, USA), following standard preprocessing steps outlined in Supplementary Material S1.1. Clean data were segmented into stimulus-locked epochs from -100 ms (pre-stimulus onset) to 600 ms (post-stimulus onset) and sorted to correct Go, correct NoGo, incorrect Go, and incorrect NoGo conditions.

Recomputed to an average reference, baseline-corrected (−100 to 0 ms pre-stimulus onset), artifact-free epochs were averaged to compute ERP waveforms for each participant. Only correct Go and correct NoGo trials with a minimum of 40 artifact-free epochs per condition were included in the final analysis. On average, participants had 60.43 ± 7.45 artifact-free epochs for Go trials and 58.47 ± 8.03 epochs for NoGo trials across a total of 133 participants.

### 2.4. ERP Components

Mean amplitudes (averaged over a 40 ms window centered on peak latency) and latencies (time points of peak amplitudes) were extracted within established time windows based on grand average ERP waveforms and prior research. The N1 peak was defined as the most negative peak between 80 and 190 ms post-stimulus; the N2 peak was defined as the most negative peak between 190 and 350 ms post-stimulus; the P2 peak was defined as the most positive peak between 150 and 270 ms post-stimulus; and the P3 peak was defined as the most positive peak between 240 and 600 ms post-stimuli. All components were measured at Fz, FCz, Cz, CPz, and Pz electrodes.

### 2.5. Questionnaires

General demographical assessment (gender, age, weight, height, handedness, and education) was conducted alongside internet usage patterns (PIUQ-9, The Nine-Item Problematic Internet Use Questionnaire (Burkauskas et al., 2020; Koronczai et al., 2011) and DPIU, The Dimensions of Problematic Internet Use (Van Ameringen et al., 2018)). The levels of anxiety (BAI, Beck Anxiety Inventory (Beck et al., 1988)), depression (BDI-II, Beck Depression Inventory, Second Edition (Beck et al., 1996)), and obsessive–compulsive symptoms (CBOCI, Clark–Beck Obsessive–Compulsive Inventory (Clark et al., 2005)) were measured.

#### 2.5.1. The Nine-Item Problematic Internet Use Questionnaire (PIUQ-9)

PIUQ-9 (Koronczai et al., 2011) is designed to measure the extent of problematic internet use among individuals and its psychometric functions were validated in a Lithuanian sample (Burkauskas et al., 2020). The PIUQ-9 is a brief version consisting of 9 items, divided into three subscales: obsession, neglect, and control disorder. Scores range from 9 to 45, with a provisional cutoff score of 22 indicating problematic use and higher scores indicating more severe involvement. The PIUQ-9 has been validated through factorial investigation and aligns with findings from previous research, making it a reliable tool for assessing problematic internet behaviors. Cronbach’s alpha of the scale in the current sample α = 0.79.

#### 2.5.2. The Dimensions of Problematic Internet Use (DPIU)

DPIU (Van Ameringen et al., 2018) is a self-report measure, assessing problematic internet use based on DSM-V criteria for addiction (American Psychiatric Association, 2013). It begins with 3 YES/NO initial screener questions, and if two or more are answered affirmatively, respondents proceed to 5 additional questions using a 6-point Likert scale with scores ranging from 0 (not at all) to 5 (a lot). The scale covers nine dimensions of problematic internet use, including entertainment and video streaming, social media, gaming, messaging, dating apps, gambling, sexual content, online shopping, and information search. Scores of 3 or more on three or more dimensions indicate potential addiction. Cronbach’s alpha values for the subscales were as follows: entertainment and video streaming α = 0.79, social media α = 0.77, gaming α = 0.88, messaging α = 0.82, dating apps α = 0.65, sexual content α = 0.68, online shopping α = 0.74, and information search α = 0.68. The Cronbach’s alpha values for some DPIU subscales, although not uniformly high, are indicative of a moderate level of internal consistency which is acceptable for exploratory research into the complex and varied patterns of problematic internet use. However, the gambling dimension was excluded due to zero participants scoring within the subscale, and the dating apps, sexual content, and online shopping dimensions were also omitted from subsequent analyses due to their small sample sizes.

#### 2.5.3. The Beck Anxiety Inventory (BAI)

BAI (Beck et al., 1988) is used to assess the intensity of anxiety symptoms. BAI consists of 21 items rated from 0 to 3 based on the individual’s experience of anxiety symptoms. The total score ranges from 0 to 63 points, with higher scores indicating greater severity of anxiety. The BAI has been extensively validated and is a reliable tool for assessing anxiety levels in clinical and non-clinical populations. Cronbach’s alpha of the scale in the current sample α = 0.86.

#### 2.5.4. Beck’s Depression Inventory (BDI-II)

BDI-II (Beck et al., 1996) is designed to assess the severity of depression and is widely applicable for research and clinical settings (García-Batista et al., 2018; Wang & Gorenstein, 2013). The inventory includes 21 items scored from 0 to 3, reflecting the frequency and intensity of depressive symptoms. The total score ranges from 0 to 63, with higher scores indicating greater severity of depression. In non-clinical populations, scores above 20 indicate depression, while in clinical populations, scores categorize the severity of depression from minimal to severe. The internal consistency of the BDI-II in the current study was excellent (α = 0.9).

#### 2.5.5. The Clark–Beck Obsessive–Compulsive Inventory (CBOCI)

CBOCI (Clark et al., 2005) measures obsessive and compulsive symptoms to identify Obsessive–Compulsive Disorder (OCD). Participants respond to 25 items scored on a four-point Likert scale that screens for obsessive and compulsive behaviors and thoughts. The CBOCI includes two subscales: 14 items on obsessions and 11 items on compulsions evaluation. Scores range from 0 to 72, with higher scores in each subscale indicating greater severity of respective symptoms. The Cronbach’s alpha values for the subscales were as follows: total CBOCI score α = 0.92, obsessions subscale α = 0.88, and compulsions subscale α = 0.86.

### 2.6. Statistical Analysis

Statistical analyses were conducted using MS Excel (version 2053, Microsoft Corporation, 2018), JASP 0.18.3 (JASP Team, 2024), and SPSS (version 29.0.2.0, IBM SPSS, n.d.). Reaction times (RT) for Go trials were measured from stimulus onset to response, with the mean RT, intra-subject RT standard deviation (SD), and response accuracy (percentage of correct Go and NoGo responses) computed.

To comprehensively investigate the relationship between internet use severity and cognitive control, two complementary analytical approaches were employed. First, a continuous evaluation across the entire sample was conducted using correlation analyses to assess the associations between PIU scores, behavioral performance, psychological variables, and ERP amplitudes and latencies (N1, N2, P2, and P3). Normality was tested using the Shapiro–Wilk test, followed by Spearman’s correlation analysis. To enhance result robustness, non-parametric bootstrapping (5000 replicates) was applied, and significant findings were corrected for multiple comparisons using False Discovery Rate (FDR) (Benjamini & Hochberg, 1995).

In parallel, a group-based approach was applied to compare individuals at the extremes of internet use. Participants were categorized into low and high PIU groups based on PIUQ-9 scores, using a quartile-based approach with inclusive thresholds. The lower threshold for the high PIU group was a score of 23, which closely aligns with the provisional cutoff score of 22 suggested in earlier research (Koronczai et al., 2011). This overlap supports the validity of using a quartile-based method in our non-clinical sample, while also capturing individuals whose PIUQ-9 scores approximate clinical relevance.

To examine ERP differences between the high and low PIU groups, Shapiro–Wilk tests were conducted to assess the normality of ERP peak amplitudes and latencies across conditions (Go/NoGo) and electrode sites (Fz, FCz, Cz, CPz, and Pz). Levene’s test was performed to evaluate the homogeneity of variance between PIU groups. As violations of normality and homogeneity of variance were detected, Mann–Whitney U tests were employed to compare ERP components between groups. The same statistical approach was applied to analyze differences in behavioral and psychological variables between groups. In case of significant results, findings were corrected for multiple comparisons using FDR (Benjamini & Hochberg, 1995).

This dual-approach analysis—continuous evaluation of PIU severity and categorical comparison of extreme groups—aimed to uncover both gradual neurophysiological changes and distinct differences associated with varying levels of internet engagement.

## 3. Results

### 3.1. Participants’ Data

Descriptive statistics for the internet usage patterns and psychological measures assessed are presented in Table 1. Due to the small number of participants reporting usage of the dating apps (n = 9), sexual content (n = 14), and online shopping (n = 18) categories, these domains were excluded from the correlational analysis.

The mean PIUQ-9 score (19.85 ± 5.32) of the whole sample falls below the provisional cutoff of 22 for problematic internet use (Koronczai et al., 2011), though individual differences suggest varying levels of severity (out of 133 participants, 43 scored 22 or above). The low PIU group (n = 35, 17 females, and PIU score 13.83 ± 1.95) included individuals with scores ranging from 10 to 16, while the high PIU group (n = 38, 24 females, and PIU score 26.76 ± 3) comprised individuals with scores from 23 to 36.

The DPIU total score (25.97 ± 18.8) reflected diverse engagement across online activities, with entertainment/video streaming (9.99 ± 4.5) and social media (9.55 ± 4.33) being the most common, while gaming (12.27 ± 5.36) and messaging (13.3 ± 5.26) showed higher variability.

Psychological measures indicated moderate group levels of anxiety (BAI: 31.71 ± 7.64), with some participants likely experiencing clinically relevant symptoms (Beck et al., 1988). The mean BDI score (10.72 ± 8.95) indicated generally low depressive symptoms in a cohort, though suggesting individual differences. The CBOCI total score (19.49 ± 11.67) highlighted variability in obsessive–compulsive traits, with obsession-related symptoms (11.99 ± 6.93) exceeding compulsions (7.54 ± 5.91).

Overall, these findings support wide-ranging internet use behaviors and different levels of experienced psychological symptoms.

### 3.2. Behavioral Results

The average reaction time in the Go trials was 425 ± 70 ms, and the accuracy of the Go condition was 96.81%, corresponding to an average of 72.61 ± 4.82 correct responses. The mean accuracy of the NoGo trials was at 96.97%, equivalent to 72.73 ± 2.53 successful inhibitions.

#### PIU Questionnaires and Behavioral Responses

No significant correlations were found between PIU scores and behavioral performance, except for a positive correlation between gaming scores and Go reaction times (rs = 0.32, *p* = 0.003), indicating that higher gaming-related internet use is linked to slower response execution.

The lack of correlation between overall PIU scores and behavioral variables suggests that internet use severity does not directly impact basic cognitive performance. Full results are available in Supplementary Material S2, Table S1.

### 3.3. PIU Questionnaires and Psychological Measures

**Figure 1:**
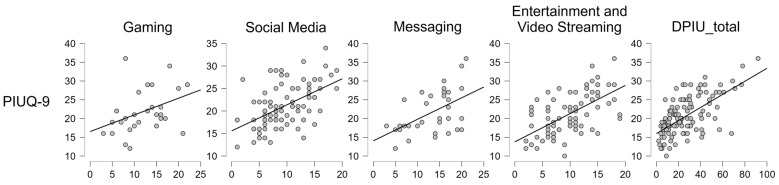
Correlation of PIUQ-9 with internet activity domains. Correlation of PIUQ-9 with gaming, social media, messaging, entertainment and video streaming, and DPIU_total scales scores.

As expected, strong correlations were found between PIUQ-9 and DPIU scores, as well as among most DPIU subdomains, confirming the consistency of problematic internet use measures. Full correlation results and visual representations for significant associations are shown in Table 2 and Figure 1.

#### Correlation Between PIU Questionnaires and Psychological Questionnaires

As expected, higher PIUQ-9 and DPIU scores were associated with poorer psychological states. PIUQ-9 scores correlated positively with (BAI, rs = 0.366, *p* < 0.001), depression (BDI, rs = 0.333, *p* < 0.001), obsessions (CBOCI obsessions subscale, rs = 0.357, *p* < 0.001), and compulsions (CBOCI compulsions subscale, rs = 0.376, *p* < 0.001) subscales, and the total score of obsessive–compulsive symptoms (total CBOCI score, rs = 0.421, and *p* < 0.001). Similarly, DPIU total scores were positively correlated with anxiety (BAI, rs = 0.359, and *p* < 0.001), depression (BDI, rs = 0.379, and *p* < 0.001), obsessive–compulsive symptoms (CBOCI, rs = 0.384, and *p* < 0.001) including both obsessions (CBOCI obsessions subscale, rs = 0.388, and *p* < 0.001) and compulsions (CBOCI compulsions subscale, rs = 0.277, and *p* = 0.002). These findings suggest that greater internet use severity is linked to higher levels of anxiety, depression, and obsessive–compulsive symptoms.

Among DPIU subdomains, social media use positively correlated with anxiety (BAI, rs = 0.301, and *p* = 0.005) and depression (BDI, rs = 0.333, and *p* = 0.002) levels. Entertainment and video streaming use was positively correlated with anxiety (BAI, rs = 0.285, and *p* = 0.009), depression (BDI, rs = 0.247, and *p* = 0.028), and obsessions (CBOCI obsessions subscale, rs = 0.251, and *p* = 0.022). Correlations of gaming, messaging and information search dimensions did not reach significance with any of psychological variables, suggesting that the impact of internet use on mental health varies across different online activities.

Detailed correlation results are available in Supplementary Material S2, Table S2, and correlation plots for significant associations are provided in Supplementary Material S2, Figure S1.

### 3.4. ERP Results

**Figure 2:**
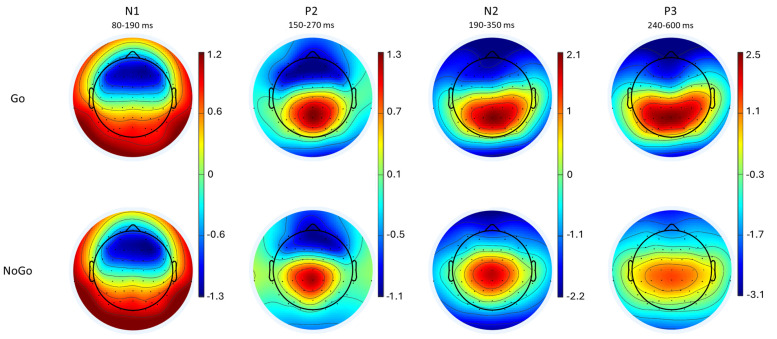
Topographical maps for Go and NoGo stimuli. Topographical representation of the N1, P2, N2, and P3 components in response to Go (**top row**) and NoGo (**bottom row**) stimuli.

Descriptive statistics for ERP amplitudes and latencies in Go and NoGo conditions are presented in Table 3. As expected, Go-P3 exhibited a more parietal distribution, while NoGo-P3 was more centrally distributed, aligning with previous studies using the equiprobable Go/NoGo task (Barry et al., 2016; Melynyte et al., 2017). The averaged topographic maps illustrating the spatial distribution of each ERP component for both conditions are shown in Figure 2.

#### 3.4.1. PIU Questionnaires and ERP Components in Go Condition

A negative correlation was observed between N1 amplitude at Cz and the gaming domain (rs = −0.467, *p* = 0.009), indicating that higher gaming-related internet use is associated with reduced N1 amplitude. A significant negative correlation was found between N1 latency at CPz and the total DPIU score (rs = −0.24, *p* = 0.009), suggesting a potential link between increased problematic internet use and faster N1 responses. Detailed statistical results are available in Supplementary Material S2, Tables S3 and S4.

#### 3.4.2. PIU Questionnaires and ERP Components in NoGo Condition

A negative correlation was observed between N1 amplitudes and the gaming domain at Cz (rs = −0.486, *p* = 0.006), suggesting that greater engagement in gaming is linked to reduced early sensory processing during inhibitory control.

Analysis of ERP latencies in NoGo trials revealed significant association between P3 latency and information search domain. Positive correlations observed within three electrode sites (Fz: rs = 0.503, *p* = 0.017; Cz: rs = 0.443, *p* = 0.039; and Pz: rs = 0.466, *p* = 0.029). This finding suggests that greater engagement in information-seeking activities is associated with delayed P3 responses during inhibitory processing, potentially reflecting increased cognitive demand or reduced efficiency in response inhibition.

These results suggest that higher levels of problematic internet use, particularly in gaming and information-seeking activities, may be linked to alterations in early sensory processing and inhibitory control mechanisms.

Detailed statistical results are provided in Supplementary Material S2, Tables S5 and S6.

#### 3.4.3. Low vs. High Engagement in PIU

The low PIU and high PIU groups did not differ in age (*p* = 0.461) but showed significant differences across all psychological measures, as detailed in Table 4. Descriptive statistics for psychological measures between the groups are presented in Supplementary Material S3, Table S7.

Despite these differences in psychological domains, no significant differences were detected at the behavioral level (Table 5). Descriptive statistics for behavioral measures between the groups are presented in Supplementary Material S3, Table S8.

**Figure 3:**
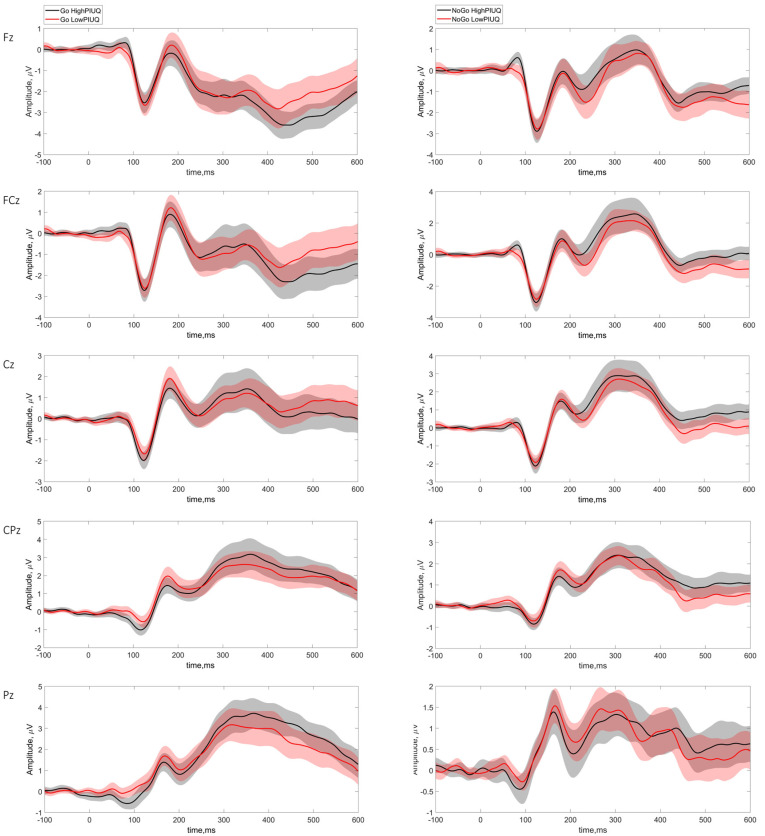
Grand average ERP waveforms for Go and NoGo stimuli across high and low internet use groups. Grand average ERP waveforms to Go (**left column**) and NoGo (**right column**) stimuli at midline electrode sites (Fz, FCz, Cz, CPz, and Pz) between high PIUQ-9 scores (black line) and low PIUQ-9 scores (red line) groups. The shaded areas around each waveform signify the 1.96×standard error (mean ± 1.96*SE).

No significant differences between the groups were detected at the neurophysiological level of Go/NoGo tasks (Table 6). The averaged ERP waveforms for Go and NoGo conditions are displayed in Figure 3 and topographical representation of the components in response to Go and NoGo stimuli between the groups is presented in Supplementary Material S3, Figure S2. Descriptive statistics for ERP amplitudes and latencies are presented in Supplementary Material S3, Table S9.

## 4. Discussion

Our findings in a healthy young adult sample revealed several significant insights across psychological, behavioral, and neurophysiological domains. First, weak to moderate correlations between PIU severity and symptoms of anxiety, depression, and obsessive–compulsive behaviors support the well-documented link between problematic internet use and psychological distress. Second, behavioral performance on the Go/NoGo task was unaffected by PIU severity, with only a slight delay in reaction times observed in higher gaming engagement. Third, at the neurophysiological level, negative correlations between the N1 component and PIU severity—particularly in the gaming domain—suggest altered early sensory processing, whereas a positive correlation between NoGo-P3 latency and information search domain suggests delayed inhibitory processing. Finally, despite significant psychological differences between high and low PIU groups, no ERP or behavioral differences were found, suggesting that neurophysiological alterations may develop gradually along the PIU continuum rather than manifesting as categorical differences.

### 4.1. Behavioral Level

As commonly reported in addiction-related research, deficits in cognitive control are often linked to problematic behaviors (Littel et al., 2012; Pandey et al., 2016; Ramey & Regier, 2019; Yao et al., 2015; Zhou et al., 2010). However, our results challenge this assumption as we found no significant correlations between PIU and behavioral performance on the Go/NoGo task. Only the gaming domain showed a positive correlation with Go reaction times, suggesting a subtle delay in response execution among individuals with higher gaming engagement. This finding may reflect increased altered motor planning rather than a generalized cognitive deficit in individuals with higher PIU.

### 4.2. Psychological Level

Consistent with previous meta-analyses and systematic reviews (Kuss & Lopez-Fernandez, 2016; Noroozi et al., 2021), our findings confirm strong associations between PIU severity and symptoms of anxiety, depression, and obsessive–compulsive behaviors. The significant psychological differences between high and low PIU groups further reinforce the well-established mental health impact of problematic internet behaviors.

However, specific PIU subdomains varied in their associations with levels of experienced depression, anxiety, and obsessive–compulsive symptoms. While social media and entertainment/video streaming were linked to anxiety and depression, entertainment/video streaming also correlated with obsessive–compulsive symptoms. In contrast, messaging and information search domains showed no significant associations with psychological measures, reinforcing the idea that different online activities contribute differently to mental health outcomes. These findings once again reinforce the notion that despite common underlying factors (Andreassen et al., 2013; Robbins & Clark, 2015), different forms of PIU might have distinct psychological underpinnings These findings align with theoretical models suggesting that PIU is not a uniform disorder but rather a spectrum of behaviors with distinct neurobiological and psychological underpinnings (Griffiths, 2014; Király et al., 2014; Schou Andreassen et al., 2016).

### 4.3. Neurophysiological Level

#### 4.3.1. Early Sensory Processing (N1 and P2)

N1 amplitudes were negatively correlated with gaming scores in both Go and NoGo conditions, while N1 latencies in the Go condition were negatively correlated with overall DPIU scores. Given that N1 reflects early sensory processing and attentional allocation (J. S. Fogarty et al., 2020; Joos et al., 2014; Tomé et al., 2015), these findings suggest that high PIU engagement—particularly in gaming—may influence how sensory information is processed at an early stage.

The observation of reduced N1 amplitudes in high-gaming individuals aligns with previous findings, such as Park et al. (2017a), who reported lower N1 amplitudes in IGD groups during an oddball task. Thus, these results align with a broader view that N1 alterations may indicate differences in stimulus-driven processing among heavy internet users rather than being a specific biomarker for IGD (Kashif et al., 2021).

Similarly to N1, P2 is considered a stimulus-related component involved in sensory and perceptual processing (J. S. Fogarty et al., 2020) and is thought to reflect more advanced perceptual processes necessary for target identification (Crowley & Colrain, 2004). However, no associations between P2 components and PIU severity or any of the internet use dimensions were observed.

#### 4.3.2. Response Monitoring and Inhibition (N2 and P3)

We found no significant correlations between N2 amplitudes and PIU measures. While some studies find lower NoGo-N2 amplitudes in PIU and IGD (Dong et al., 2010; Fathi et al., 2024; Lee & Kang, 2014; Zhou et al., 2010), others report increased or inconsistent N2 responses (Chen et al., 2018; Littel et al., 2012). Previous research also demonstrated prolonged N2 latencies related to PIU (Kim et al., 2017; Ge et al., 2011). The lack of significant N2 findings in our study suggests that attentional conflict monitoring remains intact in a non-clinical population.

A notable finding was the positive correlation between NoGo-P3 latency and the information search domain, suggesting delayed inhibitory processing in individuals who frequently engage in extensive online searching. Reductions in NoGo-P3 amplitude have been documented in substance use disorders (Colrain et al., 2011; Evans et al., 2009; Porjesz & Begleiter, 2003; Sokhadze et al., 2008; Yin et al., 2016); it was also observed in IGD (Park et al., 2016, 2017a, 2017b). Our findings indicate that P3 alterations may reflect both differences in response-related updating in working memory leading to motor activation and the generation of movement-related sensory feedback (J. Fogarty, 2020) and deficits in behavioral inhibition (Donkers & van Boxtel, 2004; Nieuwenhuis et al., 2004; Randall & Smith, 2011).

Taken together, our findings suggest that while PIU may influence early sensory processing (N1) and response inhibition (NoGo-P3), its impact on response monitoring and execution (N2 and Go-P3) is less pronounced in non-clinical populations.

#### 4.3.3. High vs. Low PIU Comparison

Despite the observed correlational patterns in the whole sample, no significant ERP or behavioral differences were found between high and low PIU groups. This suggests that while PIU severity may influence specific neurophysiological markers, its effects do not scale linearly across the PIU continuum in a healthy sample. Previous studies have consistently shown distinct ERP alterations in clinical PIU or IGD groups (Chen et al., 2018; Dong et al., 2010; Ge et al., 2011; Yu et al., 2009; Zhou et al., 2010), suggesting that neural differences may become more pronounced only in severe cases. Our findings highlight the importance of studying PIU as a continuum (Anderson et al., 2017) rather than assuming categorical differences between high and low engagement groups.

## 5. Conclusions

Our findings reinforce the associations between PIU and psychological symptoms, while showing subtle but notable alterations in early sensory processing and response inhibition at the neurophysiological level. However, the lack of significant ERP differences between high and low PIU groups suggests that measures obtained in equiprobable Go/NoGo tasks may not be highly sensitive biomarkers for PIU risk in non-clinical populations. Our findings also highlight the need to distinguish between different types and severities of internet use in the general population. Subtle ERP alterations linked to gaming and information-seeking behaviors underlines the importance of paying attention to cognitive patterns in individuals who may not meet clinical criteria but still show early signs of altered processing. In educational and mental health contexts, such insights may support timely, low-intensity interventions aimed at promoting healthier digital engagement.

## 6. Limitations

This study has several limitations. First, it focused on a healthy, non-clinical young adult population, limiting the generalizability of findings to clinical groups or individuals with severe problematic behaviors. The sample of 133 participants allowed for a broad correlational analysis, but certain internet use domains were underrepresented, reducing statistical power and limiting the reliability of findings in these areas. As a result, subscales with particularly small samples (e.g., gambling, dating apps, online shopping, and sexual content) were excluded. Future research should aim for more balanced recruitment to improve representation across all domains.

## Figures and Tables

**Table 1:** Descriptive statistics for the Internet Usage Patterns and Psychological Evaluation Measures within the sample.

Variable	Valid (n)	Mean ± SD
PIUQ9	133	19.85 ± 5.32
DPIU_total score	119	25.97 ± 18.8
DPIU_Entertainment and Video Streaming	83	9.99 ± 4.5
DPIU_Social Media	84	9.55 ± 4.33
DPIU_Gaming	30	12.27 ± 5.36
DPIU_Messaging	33	13.3 ± 5.26
DPIU_Dating Apps	9	6.78 ± 3.866
DPIU_Sexual Content	14	10.5 ± 4.18
DPIU_Online Shopping	18	8.39 ± 3.9
DPIU_Information Search	22	13.32 ± 3.82
BAI	132	31.71 ± 7.64
BDI	129	10.72 ± 8.95
CBOCI	132	19.49 ± 11.67
CBOCI_obsessions	132	11.99 ± 6.93
CBOCI_compulsions	133	7.54 ± 5.91

BAI—Beck Anxiety Inventory; BDI—Beck Depression Inventory; CBOCI—Clark–Beck Obsessive–Compulsive Inventory; DPIU—The Dimensions of Problematic Internet Use; and PIUQ-9—The Nine-Item Problematic Internet Use Questionnaire.

**Table 2:** Spearman’s Correlations between Problematic Internet Use Questionnaires (PIUQ-9 and DPIU).

Variable		PIUQ-9	Gaming	Social Media	Messaging	Entertainment and Video Streaming
Gaming	Spearman’s rho	0.426 *	—			
	*p*-value	0.019	—			
	N	30	—			
Social Media	Spearman’s rho	0.512 *	0.487 *	—		
	*p*-value	<0.001	0.029	—		
	N	84	20	—		
Messaging	Spearman’s rho	0.472 *	0.43	0.644 *	—	
	*p*-value	0.005	0.288	<0.001	—	
	N	33	8	27	—	
Entertainment and Video Streaming	Spearman’s rho	0.603 *	0.326	0.453 *	0.758 *	—
	*p*-value	<0.001	0.129	<0.001	<0.001	—
	N	83	23	56	22	—
DPIU_total	Spearman’s rho	0.585 *	0.392 *	0.59 *	0.781 *	0.622 *
	*p*-value	<0.001	0.032	<0.001	<0.001	<0.001
	N	119	30	84	33	83

* The significance of the results survived FDR correction. DPIU—The Dimensions of Problematic Internet Use and PIUQ-9—The Nine-Item Problematic Internet Use Questionnaire. The variation in sample sizes across the subscales is attributed to the low overlap between specific dimensions, resulting in smaller sample sizes for certain subscales within the study.

**Table 3:** Descriptive statistics for the amplitudes and latencies of ERP components during Go and NoGo conditions.

	Go Condition				NoGo Condition			
Electrode	N1	N2	P2	P3	N1	N2	P2	P3
Amplitudes								
Fz	−2.49 ± 1.4	−2.86 ± 2.25	0.25 ± 1.69	−1.1 ± 2.19	−2.62 ± 1.49	−1.53 ± 1.9	0.28 ± 1.8	1.55 ± 1.99
FCz	−2.48 ± 1.33	−1.78 ± 2.11	1.1 ± 1.75	0.17 ± 2.52	−2.57 ± 1.4	−0.66 ± 1.78	1.08 ± 1.77	3.06 ± 2.43
Cz	−1.7 ± 1.06	−0.38 ± 1.67	1.64 ± 1.57	1.79 ± 2.5	−1.75 ± 1.06	0.22 ± 1.36	1.5 ± 1.42	3.48 ± 2.19
CPz	−0.85 ± 0.87	0.64 ± 1.47	1.76 ± 1.31	3.33 ± 2.36	−0.85 ± 0.9	0.6 ± 1.24	1.68 ± 1.24	2.95 ± 1.66
Pz	−0.51 ± 0.87	0.67 ± 1.5	1.64 ± 1.25	4.03 ± 2.31	−0.54 ± 0.85	0.15 ± 1.44	0.15 ± 1.44	2.19 ± 1.5
Latencies								
Fz	127.12 ± 12.23	280.39 ± 43.06	191.13 ± 19.99	366.25 ± 68.7	128.44 ± 15.41	248.54 ± 34.93	189.74 ± 28.32	339.57 ± 41.2
FCz	125.25 ± 9.4	263.71 ± 33.35	188.13 ± 17.47	340.19 ± 42.83	126.03 ± 10.49	240.08 ± 31.15	188.32 ± 22.96	332.07 ± 40.74
Cz	123.37 ± 11.58	259.44 ± 35.4	189.8 ± 21.78	352.71 ± 53.42	124.24 ± 8.257	235 ± 31.96	184.79 ± 17.72	328.24 ± 43.26
CPz	115.07 ± 18.73	242.3 ± 37.25	189.14 ± 24.52	362.07 ± 61.45	117.18 ± 16.63	237.34 ± 38.36	189.9 ± 29.83	334.85 ± 53.62
Pz	97.12 ± 23.67	224.05 ± 35.94	173.57 ± 28.81	362.52 ± 60.31	99.81 ± 27.271	239.94 ± 42.87	182.9 ± 30.957	350.18 ± 63

Amplitudes are reported in microvolts (μV) and latencies are reported in milliseconds (ms); means ± SDs provided.

**Table 4:** Group differences between Psychological Evaluation Measures.

Variable	U	*p*	Effect Size	SE Effect Size
PIUQ9	1330	<0.001 *	1	0.135
BAI	997	<0.001 *	−0.544	0.136
BDI	891	0.003 *	−0.417	0.137
CBOCI	1040	<0.001 *	−0.565	0.135
CBOCI obsessions	975.5	<0.001 *	−0.467	0.135
CBOCI compulsions	1034	<0.001 *	−0.555	0.135

* The significance of the results survived FDR correction. Note. Mann–Whitney U test. BAI—Beck Anxiety Inventory; BDI—Beck Depression Inventory; CBOCI—Clark–Beck Obsessive–Compulsive Inventory, DPIU—The Dimensions of Problematic Internet Use; PIUQ-9—The Nine-Item Problematic Internet Use Questionnaire; SE effect size—standard error of effect size; and U—Mann–Whitney U test statistic.

**Table 5:** Group differences between Behavioral Measures.

Variable	U	*p*	Effect Size	SE Effect Size
Correct_Go	617	0.571	0.072	0.135
Correct_NoGo	734	0.436	−0.104	0.135
GO_RT	766	0.267	−0.152	0.135

Note. Mann–Whitney U test. GO_RT—average Go reaction time; SE effect size—standard error of effect size; and U—Mann–Whitney U test statistic.

**Table 6:** Group differences between the amplitudes and latencies of ERP components during Go condition.

		Amplitudes				Latencies			
Electrode Site	Component	U	*p*	Effect Size	SE Effect Size	U	*p*	Effect Size	SE Effect Size
		Go Condition							
Fz	N1	709	0.633	0.066	0.135	676.5	0.903	0.017	0.135
	N2	634	0.738	−0.047	0.135	574.5	0.32	−0.136	0.135
	P2	625	0.665	−0.06	0.135	604	0.504	−0.092	0.135
	P3	580	0.353	−0.128	0.135	467	0.029	−0.298	0.135
FCz	N1	652	0.891	−0.02	0.135	616.5	0.596	−0.073	0.135
	N2	622	0.641	−0.065	0.135	727.5	0.494	0.094	0.135
	P2	626	0.673	−0.059	0.135	635.5	0.749	−0.044	0.135
	P3	634	0.738	−0.047	0.135	556.5	0.233	−0.163	0.135
Cz	N1	551	0.211	−0.171	0.135	560.5	0.251	−0.157	0.135
	N2	652	0.891	−0.02	0.135	730.5	0.473	0.098	0.135
	P2	586	0.388	−0.119	0.135	734	0.449	0.104	0.135
	P3	682	0.856	0.026	0.135	738.5	0.42	0.111	0.135
CPz	N1	515	0.099	−0.226	0.135	551.5	0.212	−0.171	0.135
	N2	713	0.602	0.072	0.135	624	0.655	−0.062	0.135
	P2	542	0.177	−0.185	0.135	678	0.89	0.02	0.135
	P3	753	0.336	0.132	0.135	707.5	0.643	0.064	0.135
Pz	N1	440	0.013	−0.338	0.135	587.5	0.395	−0.117	0.135
	N2	665	1	0	0.135	498	0.066	−0.251	0.135
	P2	578	0.342	−0.131	0.135	623.5	0.65	−0.062	0.135
	P3	741	0.407	0.114	0.135	706	0.655	0.062	0.135
		**NoGo condition**							
Fz	N1	644	0.822	−0.032	0.135	689	0.795	0.036	0.135
	N2	709	0.633	0.066	0.135	685.5	0.825	0.031	0.135
	P2	659	0.952	−0.009	0.135	720.5	0.544	0.083	0.135
	P3	708	0.641	0.065	0.135	742	0.398	0.116	0.135
FCz	N1	649	0.865	−0.024	0.135	626.5	0.675	−0.058	0.135
	N2	716	0.579	0.077	0.135	709.5	0.627	0.067	0.135
	P2	719	0.557	0.081	0.135	720.5	0.544	0.083	0.135
	P3	669	0.969	0.006	0.135	692.5	0.766	0.041	0.135
Cz	N1	646	0.839	−0.029	0.135	594	0.436	−0.107	0.135
	N2	664	0.996	−0.002	0.135	664	0.996	−0.002	0.135
	P2	659	0.952	−0.009	0.135	680	0.873	0.023	0.135
	P3	697	0.73	0.048	0.135	673	0.934	0.012	0.135
CPz	N1	619	0.617	−0.069	0.135	672.5	0.938	0.011	0.135
	N2	575	0.325	−0.135	0.135	683	0.847	0.027	0.135
	P2	622	0.641	−0.065	0.135	675.5	0.912	0.016	0.135
	P3	662	0.978	−0.005	0.135	813	0.103	0.223	0.135
Pz	N1	632	0.721	−0.05	0.135	773.5	0.231	0.163	0.135
	N2	578	0.342	−0.131	0.135	794	0.156	0.194	0.135
	P2	588	0.401	−0.116	0.135	654.5	0.912	−0.016	0.135
	P3	603	0.499	−0.093	0.135	752.5	0.337	0.132	0.135

No results survived FDR correction. Note. Mann−Whitney U test. SE effect size—standard error of effect size; and U—Mann−Whitney U test statistics.

## Data Availability

The data presented in this study are available on request from the corresponding author.

## Supplementary Materials

The following supporting information can be downloaded at: https://www.mdpi.com/article/10.3390/bs15050579/s1. Supplementary Material S2: Table S1: Spearman’s Correlations between Problematic Internet Use Questionnaires (PIUQ-9 and DPIU) and Behavioral Responses; Table S2: Spearman’s Correlations between Psychological Evaluation Measures and Problematic Internet Use Questionnaires (PIUQ-9 and DPIU); Figure S1: Correlations plots of PIUQ-9, DPIU, DPIU_Entertainment and Video Streaming, DPIU_Social media and DPIU_Gaming scales scores with psychological evaluation questionnaires scores; Table S3: Spearman’s Correlations between Internet Use Questionnaires (PIUQ-9 and DPIU) and N1, N2, P2 and P3 amplitudes during Go trials; Table S4: Spearman’s Correlations between Internet Use Questionnaires (PIUQ-9 and DPIU) and N1, N2, P2 and P3 latencies during Go trials; Table S5: Spearman’s Correlations between Internet Use Questionnaires (PIUQ-9 and DPIU) and N1, N2, P2 and P3 amplitudes during NoGo trials; Table S6: Spearman’s Correlations between Internet Use Questionnaires (PIUQ-9 and DPIU) and N1, N2, P2 and P3 latencies during NoGo trials. Supplementary Material S3: Table S7: Descriptive statistics for Psychological Measures between High and Low Internet Use involvement groups; Table S8: Descriptive statistics for Behavioral Responses between High and Low Internet Use involvement groups; Table S9: Descriptive statistics for the amplitudes and latencies of ERP components during Go and NoGo conditions within the High and Low PIU engagement groups; Figure S2: Topographical representation of the N1 (first column), P2 (second column), N2 (third column), and P3 (fourth column) waves in response to Go (upper rows) and NoGo (lower rows) stimuli between High PIU and Low PIU groups. Reference (Perrin et al., 1989) is cited in the supplementary materials.

## Abbreviations

The following abbreviations are used in this manuscript:

PIU	Problematic Internet Use
ERP	Event-Related Potential
