# Supplementary material for: Assessing the Spectrum of Internet Use in a Healthy Sample: Altered Psychological States and Intact Brain Responses to an Equiprobable Go/NoGo Task
Source: Behav Sci (Basel). 2025 Apr 25;15(5):579. doi: 10.3390/bs15050579 (PMC12109041; doi:10.3390/bs15050579)
Supplement: Supplementary file 1 [file behavsci-15-00579-s001.zip › behavsci-3546036-supplementary.pdf]

Supplementary Material S1

1.1. EEG recording and processing

EEG data processing was conducted offline using the EEGLAB toolbox within the MATLAB (The Mathworks, Natick, USA). Initial steps included the removal of power line noise utilizing the CleanLine plugin in EEGLAB, which applies Thomas F-statistics for 50Hz noise elimination. Channels with excessive artefacts underwent manual rejection, with an average of 5±2.9 channels being discarded and subsequently reconstructed through the spherical spline method (Perrin et al., 1989). Artefacts attributable to eye movements (both vertical and horizontal) and cardiac activity were corrected using ICA approach, with any remaining artefacts being manually rejected following visual inspection. Datasets were filtered between 0.1 and 25 Hz using Butterworth filter of 2nd order. Data were segmented into stimulus-locked epochs starting at -100 ms pre-stimulus onset to 600 ms post-stimulus onset and sorted to correct Go, correct NoGo, incorrect Go, and incorrect NoGo sets. The datasets were recomputed to an average reference and underwent baseline correction (-100 to 0 ms). After a thorough visual inspection, the remaining artefact-free epochs were averaged to compute average ERP waveforms for each individual participant. Trials with incorrect responses were automatically detected and excluded and only the correct Go and correct NoGo trials with a minimum number of 40 artifact-free epochs of each condition were extracted for further data analysis, with averages of 60.43±7.45 epochs for Go and 58.47±8.03 epochs for NoGo condition across 133 participants.

Supplementary Material S2

Table S1. Spearman's Correlations between Problematic Internet Use Questionnaires (PIUQ-9 and DPIU) and Behavioral Responses.

Variable		Correct_Go	Correct_NoGo	Go_RT
PIUQ-9	Spearman's rho	-0.045	0.044	0.082
	p-value	0.607	0.617	0.348
	n	133	133	133
Entertainment and Video Streaming	Spearman's rho	-0.009	0.255	0.112
	p-value	0.937	0.02	0.312
	n	83	83	83
Gaming	Spearman's rho	-0.101	0.101	0.32*
	p-value	0.358	0.359	0.003
	n	84	84	84
Social Media	Spearman's rho	0.033	0.101	0.333
	p-value	0.861	0.596	0.072
	n	30	30	30
Messaging	Spearman's rho	0.018	0.212	0.004
	p-value	0.921	0.235	0.983
	n	33	33	33
Information Search	Spearman's rho	0.043	-0.451	0.063
	p-value	0.848	0.035	0.779
	n	22	22	22
DPIU_total	Spearman's rho	0.046	0.087	0.0007
	p-value	0.622	0.345	0.994
	n	119	119	119

* The significance of the results survived FDR correction.

PIUQ-9 – The Nine-Item Problematic Internet Use Questionnaire, DPIU – The Dimensions of Problematic Internet Use, and Go_RT – average Go reaction time.

Table S2. Spearman's Correlations between Psychological Evaluation Measures and Problematic Internet Use Questionnaires (PIUQ-9 and DPIU).

Variable		BAI	BDI	CBOCI	CBOCI obsessions	CBOCI compulsions
PIUQ-9	Spearman's rho	0.366*	0.333*	0.421*	0.357*	0.376*
	p-value	< .001	< .001	< .001	< .001	< .001
	n	132	129	132	132	133
Entertainment and Video Streaming	Spearman's rho	0.285*	0.247*	0.219	0.251*	0.146
	p-value	0.009	0.028	0.047	0.022	0.189
	n	83	79	83	83	83
Gaming	Spearman's rho	0.135	0.206	0.387	0.328	0.339
	p-value	0.476	0.275	0.034	0.077	0.067
	n	30	30	30	30	30
Social Media	Spearman's rho	0.301*	0.333*	0.233	0.21	0.214
	p-value	0.005	0.002	0.034	0.056	0.05
	n	84	81	83	83	84
Messaging	Spearman's rho	0.016	0.24	0.186	0.245	0.041
	p-value	0.929	0.193	0.301	0.17	0.822
	n	33	31	33	33	33
Information Search	Spearman's rho	-0.232	0.229	0.083	0.162	-0.072
	p-value	0.299	0.331	0.713	0.472	0.749
	n	22	20	22	22	22
DPIU_total	Spearman's rho	0.359*	0.379*	0.384*	0.388*	0.277*
	p-value	< .001	< .001	< .001	< .001	0.002
	n	119	115	118	118	119

* The significance of the results survived FDR correction.

PIUQ-9 – The Nine-Item Problematic Internet Use Questionnaire, BAI – The Beck Anxiety Inventory, BDI-II – Beck's Depression Inventory, BIS-11 – The Barratt Impulsiveness Scale, CBOCI – The Clark–Beck Obsessive–Compulsive Inventory, and DPIU – The Dimensions of Problematic Internet Use.

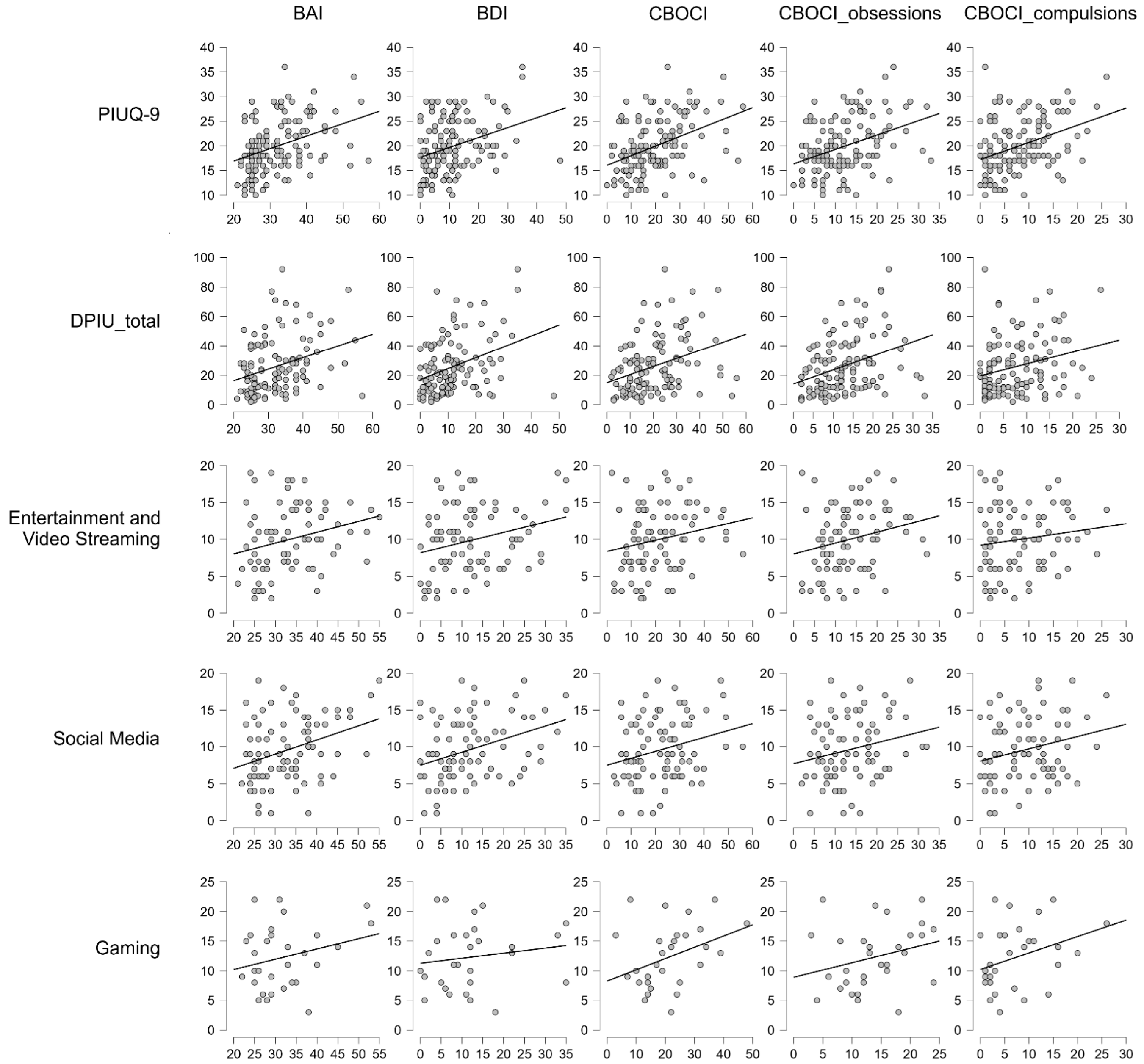

Figure S1. Correlations plots of PIUQ-9, DPIU, DPIU_Entertainment and Video Streaming, DPIU_Social media, and DPIU_Gaming scales scores with psychological evaluation questionnaires scores.
PIUQ-9 – The Nine-Item Problematic Internet Use Questionnaire, BAI – The Beck Anxiety Inventory, BDI-II – Beck's Depression Inventory, CBOCI – The Clark–Beck Obsessive–Compulsive Inventory, and DPIU – The Dimensions of Problematic Internet Use.

Table S3. Spearman's Correlations between Internet Use Questionnaires (PIUQ-9 and DPIU) and N1, N2, P2, and P3 amplitudes during Go trials.

**Go trials amplitudes**		PIUQ-9	Entertainment and Video Streaming	Social Media	Gaming	Messaging	Information Search	DPIU_total
Fz_N1	Spearman's rho	0.00015	0.114	0.17	-0.172	0.108	0.129	-0.11
	p-value	0.999	0.305	0.123	0.364	0.551	0.567	0.234
	n	133	83	84	30	33	22	119
Fz_N2	Spearman's rho	-0.084	-0.007	0.059	0.011	0.087	0.315	-0.044
	p-value	0.336	0.95	0.592	0.955	0.629	0.153	0.631
	n	133	83	84	30	33	22	119
Fz_P2	Spearman's rho	-0.069	0.036	-99.47	0.015	0.2	0.329	0.022
	p-value	0.428	0.748	0.993	0.939	0.264	0.135	0.812
	n	133	83	84	30	33	22	119
Fz_P3	Spearman's rho	-0.132	-0.13	-0.067	-0.125	-0.043	0.18	-0.145
	p-value	0.131	0.243	0.544	0.51	0.812	0.422	0.115
	n	133	83	84	30	33	22	119
FCz_N1	Spearman's rho	-0.075	0.058	0.09	-0.29	-0.055	-0.139	-0.177
	p-value	0.393	0.601	0.413	0.12	0.762	0.538	0.054
	n	133	83	84	30	33	22	119
FCz_N2	Spearman's rho	-0.075	-0.039	-0.065	-0.127	-0.069	0.109	0.001
	p-value	0.389	0.727	0.559	0.502	0.702	0.63	0.989
	n	133	83	84	30	33	22	119
FCz_P2	Spearman's rho	-0.039	0.047	-0.03	-0.067	0.178	0.176	0.015
	p-value	0.654	0.674	0.786	0.726	0.323	0.434	0.875
	n	133	83	84	30	33	22	119
FCz_P3	Spearman's rho	-0.069	-0.11	-0.112	-0.091	0.035	0.268	-0.033
	p-value	0.43	0.322	0.309	0.632	0.846	0.227	0.719
	n	133	83	84	30	33	22	119
Cz_N1	Spearman's rho	-0.147	-0.048	-0.03	-0.467*	-0.209	-0.242	-0.182
	p-value	0.091	0.668	0.785	0.009	0.244	0.278	0.047
	n	133	83	84	30	33	22	119
Cz_N2	Spearman's rho	-0.029	-0.04	-0.186	-0.182	-0.047	0.023	-0.005
	p-value	0.737	0.718	0.091	0.337	0.794	0.919	0.961
	n	133	83	84	30	33	22	119
Cz_P2	Spearman's rho	-0.042	-0.057	-0.08	-0.245	0.081	0.13	-0.007
	p-value	0.627	0.611	0.471	0.191	0.654	0.564	0.938
	n	133	83	84	30	33	22	119
Cz_P3	Spearman's rho	-0.016	-0.055	-0.138	-0.115	0.189	0.261	0.008
	p-value	0.857	0.623	0.21	0.544	0.293	0.24	0.931
	n	133	83	84	30	33	22	119
CPz_N1	Spearman's rho	-0.132	-0.2	-0.133	-0.419	-0.126	-0.141	-0.219
	p-value	0.129	0.069	0.227	0.021	0.486	0.531	0.017
	n	133	83	84	30	33	22	119
CPz_N2	Spearman's rho	0.065	-0.041	-0.109	-0.216	0.078	0.048	0.005
	p-value	0.46	0.713	0.324	0.251	0.665	0.831	0.954
	n	133	83	84	30	33	22	119
CPz_P2	Spearman's rho	-0.064	-0.147	-0.085	-0.244	0.033	-0.05	-0.03
	p-value	0.462	0.185	0.442	0.195	0.855	0.827	0.744
	n	133	83	84	30	33	22	119

Go trials amplitudes		PIUQ-9	Entertainment and Video Streaming	Social Media	Gaming	Messaging	Information Search	DPIU_total
CPz_P3	Spearman's rho	0.069	0.048	-0.131	-0.113	0.246	0.216	0.104
	p-value	0.428	0.666	0.234	0.552	0.167	0.333	0.261
	n	133	83	84	30	33	22	119
Pz_N1	Spearman's rho	-0.176	-0.219	-0.181	-0.201	-0.047	0.121	-0.203
	p-value	0.043	0.047	0.099	0.287	0.795	0.592	0.026
	n	133	83	84	30	33	22	119
Pz_N2	Spearman's rho	0.066	-0.102	-0.113	-0.203	-0.013	0.041	-0.018
	p-value	0.453	0.359	0.307	0.282	0.944	0.855	0.849
	n	133	83	84	30	33	22	119
Pz_P2	Spearman's rho	-0.021	-0.069	-0.103	-0.121	-0.164	-0.051	-0.003
	p-value	0.807	0.538	0.352	0.524	0.361	0.821	0.974
	n	133	83	84	30	33	22	119
Pz_P3	Spearman's rho	0.121	0.063	-0.092	-0.045	0.143	-0.067	0.14
	p-value	0.166	0.572	0.407	0.814	0.427	0.768	0.128
	n	133	83	84	30	33	22	119

* The significance of the results survived FDR correction.

The variation in sample sizes across the subscales is attributed to the low overlap between specific dimensions, resulting in smaller sample sizes for certain subscales within the study.

Table S4. Spearman's Correlations between Internet Use Questionnaires (PIUQ-9 and DPIU) and N1, N2, P2, and P3 latencies during Go trials.

Go trials latencies		PIUQ-9	Entertainment and Video Streaming	Social Media	Gaming	Messaging	Information Search	DPIU_total
Fz_N1	Spearman's rho	-0.032	0.046	0.094	-0.016	-0.115	0.042	-0.068
	p-value	0.712	0.681	0.394	0.931	0.524	0.852	0.46
	n	133	83	84	30	33	22	119
Fz_N2	Spearman's rho	-0.101	-0.082	-0.026	-0.233	0.085	-0.048	0.007
	p-value	0.247	0.461	0.811	0.216	0.637	0.831	0.944
	n	133	83	84	30	33	22	119
Fz_P2	Spearman's rho	-0.095	-0.128	-0.107	0.146	-0.119	0.089	-0.064
	p-value	0.276	0.25	0.331	0.441	0.511	0.695	0.491
	n	133	83	84	30	33	22	119
Fz_P3	Spearman's rho	-0.198	-0.134	-0.196	-0.416	-0.177	-0.467	-0.14
	p-value	0.023	0.227	0.074	0.022	0.324	0.028	0.13
	n	133	83	84	30	33	22	119
FCz_N1	Spearman's rho	-0.088	-0.032	0.018	-0.144	-0.17	0.086	-0.162
	p-value	0.316	0.772	0.868	0.449	0.344	0.702	0.079
	n	133	83	84	30	33	22	119
FCz_N2	Spearman's rho	0.023	0.061	0.012	0.247	0.214	-0.062	-0.04
	p-value	0.792	0.584	0.911	0.187	0.233	0.782	0.663
	n	133	83	84	30	33	22	119
FCz_P2	Spearman's rho	-0.094	-0.003	-0.017	0.097	-0.023	0.065	-0.055

**Go trials latencies**		PIUQ-9	Entertainment and Video Streaming	Social Media	Gaming	Messaging	Information Search	DPIU_total
	p-value	0.284	0.981	0.877	0.61	0.898	0.775	0.554
	n	133	83	84	30	33	22	119
FCz_P3	Spearman's rho	-0.129	-0.142	-0.173	-0.072	0.134	0.013	-0.146
	p-value	0.138	0.2	0.116	0.707	0.458	0.953	0.113
	n	133	83	84	30	33	22	119
Cz_N1	Spearman's rho	-0.126	-0.178	-0.003	-0.127	-0.178	-0.006	-0.151
	p-value	0.149	0.108	0.979	0.504	0.321	0.98	0.101
	n	133	83	84	30	33	22	119
Cz_N2	Spearman's rho	0.037	0.125	0.135	0.101	0.16	0.01	0.064
	p-value	0.671	0.261	0.222	0.594	0.375	0.966	0.486
	n	133	83	84	30	33	22	119
Cz_P2	Spearman's rho	-0.022	-0.02	-0.051	-0.097	0.035	-0.209	-0.044
	p-value	0.799	0.859	0.645	0.609	0.848	0.351	0.633
	n	133	83	84	30	33	22	119
Cz_P3	Spearman's rho	0.039	-0.007	0.122	0.003	0.094	0.393	0.027
	p-value	0.653	0.948	0.27	0.986	0.602	0.071	0.77
	n	133	83	84	30	33	22	119
CPz_N1	Spearman's rho	-0.117	-0.139	-0.137	-0.001	-0.049	0.203	-0.24*
	p-value	0.18	0.212	0.214	0.996	0.786	0.366	0.009
	n	133	83	84	30	33	22	119
CPz_N2	Spearman's rho	-0.102	-0.113	0.069	-0.027	0.109	0.046	-0.086
	p-value	0.241	0.308	0.531	0.887	0.544	0.84	0.351
	n	133	83	84	30	33	22	119
CPz_P2	Spearman's rho	-0.054	-0.132	-0.003	-0.035	0.037	0.115	-0.043
	p-value	0.534	0.234	0.976	0.852	0.838	0.61	0.643
	n	133	83	84	30	33	22	119
CPz_P3	Spearman's rho	0.001	0.058	0.165	0.151	0.077	0.13	0.093
	p-value	0.987	0.603	0.134	0.427	0.672	0.564	0.316
	n	133	83	84	30	33	22	119
Pz_N1	Spearman's rho	-0.057	-0.062	-0.102	-0.142	0.16	0.002	-0.155
	p-value	0.514	0.577	0.355	0.453	0.372	0.994	0.092
	n	133	83	84	30	33	22	119
Pz_N2	Spearman's rho	-0.171	-0.102	0.02	-0.1	-0.075	0.097	-0.117
	p-value	0.049	0.357	0.856	0.598	0.68	0.669	0.206
	n	133	83	84	30	33	22	119
Pz_P2	Spearman's rho	-0.019	-0.063	-0.004	-0.268	0.103	0.22	-0.096
	p-value	0.825	0.572	0.971	0.152	0.567	0.326	0.297
	n	133	83	84	30	33	22	119
Pz_P3	Spearman's rho	0.031	0.146	0.187	0.041	0.108	0.243	0.128
	p-value	0.725	0.188	0.089	0.829	0.548	0.276	0.167
	n	133	83	84	30	33	22	119

* The significance of the results survived FDR correction.

The variation in sample sizes across the subscales is attributed to the low overlap between specific dimensions, resulting in smaller sample sizes for certain subscales within the study.

Table S5. Spearman's Correlations between Internet Use Questionnaires (PIUQ-9 and DPIU) and N1, N2, P2, and P3 amplitudes during NoGo trials.

NoGo trials amplitudes		PIUQ-9	Entertainment and Video Streaming	Social Media	Gaming	Messaging	Information Search	DPIU_total
Fz_N1	Spearman's rho	-0.047	-0.058	0.147	-0.204	-0.153	0.073	-0.138
	p-value	0.59	0.602	0.183	0.28	0.394	0.746	0.135
	n	133	83	84	30	33	22	119
Fz_N2	Spearman's rho	0.02	0.037	0.04	0.129	0.011	-0.115	0.036
	p-value	0.819	0.737	0.718	0.496	0.952	0.612	0.697
	n	133	83	84	30	33	22	119
Fz_P2	Spearman's rho	-0.03	-0.042	0.043	0.083	-0.015	0.153	0.024
	p-value	0.734	0.709	0.701	0.663	0.934	0.498	0.793
	n	133	83	84	30	33	22	119
Fz_P3	Spearman's rho	0.015	0.018	-0.021	0.223	0.105	-0.036	0.05
	p-value	0.862	0.871	0.849	0.235	0.563	0.873	0.591
	n	133	83	84	30	33	22	119
FCz_N1	Spearman's rho	-0.071	-0.049	0.095	-0.324	-0.076	0.005	-0.174
	p-value	0.419	0.658	0.392	0.08	0.674	0.984	0.058
	n	133	83	84	30	33	22	119
FCz_N2	Spearman's rho	0.028	0.041	-0.022	0.21	0.03	0.11	0.108
	p-value	0.747	0.713	0.839	0.266	0.868	0.626	0.243
	n	133	83	84	30	33	22	119
FCz_P2	Spearman's rho	0.013	0.022	0.039	0.167	0.174	0.169	0.086
	p-value	0.886	0.846	0.727	0.378	0.332	0.451	0.354
	n	133	83	84	30	33	22	119
FCz_P3	Spearman's rho	0.019	-0.088	-0.163	0.039	0.128	0.096	0.107
	p-value	0.829	0.428	0.139	0.838	0.478	0.67	0.248
	n	133	83	84	30	33	22	119
Cz_N1	Spearman's rho	-0.066	-0.107	-0.042	-0.486*	-0.164	-0.163	-0.169
	p-value	0.45	0.337	0.702	0.006	0.363	0.467	0.067
	n	133	83	84	30	33	22	119
Cz_N2	Spearman's rho	0.009	0.003	-0.131	0.052	0.156	0.304	0.138
	p-value	0.92	0.976	0.236	0.786	0.386	0.169	0.134
	n	133	83	84	30	33	22	119
Cz_P2	Spearman's rho	-0.01	-0.007	-0.002	-0.061	0.197	0.235	0.064
	p-value	0.912	0.947	0.984	0.749	0.271	0.292	0.49
	n	133	83	84	30	33	22	119
Cz_P3	Spearman's rho	0.047	-0.083	-0.207	-0.068	0.153	-0.045	0.136
	p-value	0.589	0.458	0.059	0.722	0.395	0.841	0.139
	n	133	83	84	30	33	22	119
CPz_N1	Spearman's rho	-0.025	-0.158	-0.117	-0.549*	-0.08	-0.098	-0.124
	p-value	0.778	0.154	0.291	0.002	0.66	0.665	0.179
	n	133	83	84	30	33	22	119
CPz_N2	Spearman's rho	-0.044	-0.057	-0.186	-0.311	-0.042	0.134	0.029
	p-value	0.613	0.609	0.09	0.094	0.816	0.552	0.755
	n	133	83	84	30	33	22	119
CPz_P2	Spearman's rho	-0.025	-0.015	-0.172	-0.057	0.108	0.169	0.026
	p-value	0.774	0.895	0.119	0.766	0.551	0.451	0.779
	n	133	83	84	30	33	22	119

NoGo trials amplitudes		PIUQ-9	Entertainment and Video Streaming	Social Media	Gaming	Messaging	Information Search	DPIU_total
CPz_P3	Spearman's rho	0.053	-0.082	-0.221	-0.022	-87.89	0.176	0.067
	p-value	0.545	0.459	0.043	0.908	0.996	0.433	0.471
	n	133	83	84	30	33	22	119
Pz_N1	Spearman's rho	-0.021	-0.05	-0.078	-0.344	0.042	0.089	-0.089
	p-value	0.81	0.655	0.479	0.063	0.817	0.693	0.335
	n	133	83	84	30	33	22	119
Pz_N2	Spearman's rho	-0.067	-0.121	-0.189	-0.306	-0.176	0.121	-0.125
	p-value	0.445	0.275	0.085	0.101	0.326	0.592	0.177
	n	133	83	84	30	33	22	119
Pz_P2	Spearman's rho	-0.064	-0.058	-0.055	-0.053	0.013	0.219	-0.023
	p-value	0.465	0.604	0.622	0.779	0.942	0.328	0.803
	n	133	83	84	30	33	22	119
Pz_P3	Spearman's rho	-0.011	-0.082	-0.223*	0.041	-0.127	0.085	-0.053
	p-value	0.899	0.459	0.041	0.831	0.483	0.708	0.567
	n	133	83	84	30	33	22	119

* The significance of the results survived FDR correction.

The variation in sample sizes across the subscales is attributed to the low overlap between specific dimensions, resulting in smaller sample sizes for certain subscales within the study.

Table S6. Spearman's Correlations between Internet Use Questionnaires (PIUQ-9 and DPIU) and N1, N2, P2, and P3 latencies during NoGo trials.

NoGo trials latencies		PIUQ-9	Entertainment and Video Streaming	Social Media	Gaming	Messaging	Information Search	DPIU_total
Fz_N1	Spearman's rho	-0.023	0.124	-0.007	0.328	0.197	-0.111	-0.016
	p-value	0.789	0.265	0.952	0.076	0.272	0.622	0.865
	n	133	83	84	30	33	22	119
Fz_N2	Spearman's rho	-0.008	0.025	0.229	0.204	0.019	0.051	0.049
	p-value	0.923	0.823	0.036	0.281	0.918	0.821	0.599
	n	133	83	84	30	33	22	119
Fz_P2	Spearman's rho	0.042	0.122	0.074	0.365	0.156	-0.17	0.087
	p-value	0.63	0.273	0.504	0.047	0.386	0.45	0.349
	n	133	83	84	30	33	22	119
Fz_P3	Spearman's rho	0.024	-0.024	0.071	0.029	-0.007	0.503*	-0.022
	p-value	0.783	0.828	0.519	0.878	0.967	0.017	0.813
	n	133	83	84	30	33	22	119
FCz_N1	Spearman's rho	-0.074	-0.011	-0.103	0.101	-0.167	0.064	-0.128
	p-value	0.397	0.923	0.352	0.595	0.352	0.778	0.165
	n	133	83	84	30	33	22	119
FCz_N2	Spearman's rho	-0.016	0.039	0.196	0.433	0.17	0.133	0.018
	p-value	0.858	0.723	0.074	0.017	0.344	0.555	0.843
	n	133	83	84	30	33	22	119
FCz_P2	Spearman's rho	0.025	0.123	0.11	0.378	0.206	-0.019	0.14

**NoGo trials latencies**		PIUQ-9	Entertainment and Video Streaming	Social Media	Gaming	Messaging	Information Search	DPIU_total
	p-value	0.779	0.267	0.319	0.04	0.25	0.933	0.128
	n	133	83	84	30	33	22	119
FCz_P3	Spearman's rho	-0.049	-0.062	0.047	0.03	0.002	0.467*	-0.121
	p-value	0.578	0.578	0.669	0.876	0.993	0.028	0.188
	n	133	83	84	30	33	22	119
Cz_N1	Spearman's rho	-0.052	-0.067	-0.173	0.065	-0.091	0.474	-0.159
	p-value	0.549	0.546	0.117	0.733	0.614	0.026	0.084
	n	133	83	84	30	33	22	119
Cz_N2	Spearman's rho	-0.03	-0.009	0.115	0.258	0.2	0.405	-0.063
	p-value	0.729	0.938	0.296	0.168	0.264	0.061	0.495
	n	133	83	84	30	33	22	119
Cz_P2	Spearman's rho	-0.005	0.032	0.001	0.22	0.183	0.388	0.033
	p-value	0.954	0.774	0.993	0.244	0.308	0.074	0.719
	n	133	83	84	30	33	22	119
Cz_P3	Spearman's rho	-0.028	-0.029	0.144	0.094	0.059	0.443*	-0.12
	p-value	0.75	0.795	0.192	0.623	0.745	0.039	0.195
	n	133	83	84	30	33	22	119
CPz_N1	Spearman's rho	0.042	-0.099	0.165	-0.056	0.01	0.333	-0.046
	p-value	0.632	0.372	0.133	0.768	0.958	0.13	0.622
	n	133	83	84	30	33	22	119
CPz_N2	Spearman's rho	-0.046	-0.099	0.09	-0.018	0.226	-0.018	-0.065
	p-value	0.601	0.373	0.414	0.924	0.206	0.935	0.483
	n	133	83	84	30	33	22	119
CPz_P2	Spearman's rho	-0.048	-0.025	-0.107	-0.023	0.096	0.165	-0.037
	p-value	0.586	0.821	0.334	0.904	0.595	0.462	0.693
	n	133	83	84	30	33	22	119
CPz_P3	Spearman's rho	0.099	-0.085	0.125	0.169	0.153	0.256	-0.038
	p-value	0.257	0.442	0.256	0.372	0.395	0.251	0.68
	n	133	83	84	30	33	22	119
Pz_N1	Spearman's rho	0.055	-0.039	0.14	-0.033	-0.168	-0.145	-0.048
	p-value	0.53	0.725	0.203	0.861	0.349	0.521	0.602
	n	133	83	84	30	33	22	119
Pz_N2	Spearman's rho	0.121	-0.025	0.073	-0.151	0.437	0.422	-0.007
	p-value	0.165	0.825	0.508	0.427	0.011	0.051	0.943
	n	133	83	84	30	33	22	119
Pz_P2	Spearman's rho	-0.025	-0.074	-0.114	-0.383	-0.172	-0.057	-0.16
	p-value	0.776	0.506	0.301	0.037	0.338	0.801	0.083
	n	133	83	84	30	33	22	119
Pz_P3	Spearman's rho	0.058	-0.021	0.175	-0.059	0.127	0.466*	-0.023
	p-value	0.504	0.852	0.111	0.757	0.48	0.029	0.806
	n	133	83	84	30	33	22	119

* The significance of the results survived FDR correction.

The variation in sample sizes across the subscales is attributed to the low overlap between specific dimensions, resulting in smaller sample sizes for certain subscales within the study.

Supplementary Material S3

Table S7. Descriptive statistics for Psychological Measures between High and Low Internet Use involvement groups.

Variable	Group	n	Mean ± SD	Coefficient of Variation
PIUQ9	Low_PIU	35	13.83 ± 1.95	0.141
	High_PIU	38	26.76 ± 3	0.112
BAI	Low_PIU	34	28.59 ± 6.35	0.222
	High_PIU	38	35.74 ± 8.07	0.226
BDI	Low_PIU	34	6.97 ± 6.08	0.873
	High_PIU	37	13.3 ± 9.49	0.713
CBOCI	Low_PIU	35	13.83 ± 8.88	0.642
	High_PIU	38	25.82 ± 12.73	0.493
CBOCI obsessions	Low_PIU	35	9.54 ± 5.62	0.589
	High_PIU	38	15.37 ± 7.33	0.477
CBOCI compulsions	Low_PIU	35	4.4 ± 4.22	0.960
	High_PIU	38	10.45 ± 6.7	0.641

BAI – Beck Anxiety Inventory; BDI – Beck Depression Inventory; CBOCI – Clark–Beck Obsessive–Compulsive Inventory; DPIU – The Dimensions of Problematic Internet Use; and PIUQ-9 – The Nine-Item Problematic Internet Use Questionnaire.

Table S8. Descriptive statistics for Behavioral Responses between High and Low Internet Use involvement groups.

Variable	Group	n	Mean ± SD	Coefficient of variation
Correct_Go	Low_PIU	35	73.343± 2.838	0.039
	High_PIU	38	72.053± 6.514	0.090
Correct_NoGo	Low_PIU	35	72.714± 2.729	0.038
	High_PIU	38	72.947± 2.427	0.033
GO_RT	Low_PIU	35	0.406± 0.054	0.333
	High_PIU	38	0.424± 0.067	0.157

GO_RT – average Go reaction time.

Table S9. Descriptive statistics for the amplitudes and latencies of ERP components during Go and NoGo conditions within the High and Low PIU engagement groups.

		Go condition				NoGo condition			
Electrode site	Group	N1	N2	P2	P3	N1	N2	P2	P3
Amplitudes									
Fz	Low	-2.51±1.43	-3.04±2.47	0.3±1.98	-0.91±2.1	-2.61±1.43	-1.89±2.41	0.15±2.04	1.44±1.72
	High	-2.27±1.46	-3.22±1.98	0.03±1.82	-1.37±1.99	-2.63±1.49	-1.51±1.7	0.28±1.99	1.75±2.14
FCz	Low	-2.34±1.38	-1.76±2.52	1.15±1.88	0.21±2.26	-2.5±1.44	-1.05±2.23	0.84±1.96	2.93±1.9
	High	-2.39±1.46	-2.05±2.05	1±1.89	0.27±3	-2.59±1.5	-0.65±1.72	1.29±1.96	3.37±3.11
Cz	Low	-1.43±1.05	-0.27±1.68	1.83±1.64	1.82±2.06	-1.65±1.13	0.08±1.35	1.47±1.45	3.31±1.63
	High	-1.76±1.12	-0.43±1.7	1.53±1.49	2.12±2.92	-1.76±1.16	0.16±1.38	1.49±1.33	3.78±2.67
CPz	Low	-0.68±0.8	0.65±1.69	2.09±1.41	3.24±1.9	-0.74±0.89	0.69±1.04	1.8±1.03	2.92±1.4
	High	-1.05±0.9	0.76±1.5	1.65±1.34	3.8±2.59	-0.87±0.86	0.52±1.43	1.7±1.26	3.06±1.78
Pz	Low	-0.31±0.97	0.7±1.82	1.92±1.43	3.96±2.18	-0.47±0.66	0.3±1.33	1.81±1.09	2.23±1.44
	High	-0.8±0.92	0.71±1.63	1.57±1.28	4.36±2.24	-0.63±1.01	0.03±1.61	1.65±1.48	2.03±1.38
Latencies									
Fz	Low	125.81±13.89	284.6±46.61	188.78±14.62	381.22±65.29	126.339±16.23	248.186±32.12	187.486±27.29	334.738±40.16
	High	126.75±9.2	274.94±38.7	188.64±21.7	353.39±73.12	130.461±15.35	251.735±36.91	193.128±29.77	344.213±43.05
FCz	Low	125.15±8.42	259.42±32.68	185.39±12.4	345.82±41.53	125.112±11.16	236.18±24.5	186.11±18.12	331.61±38.33
	High	124.64±11.58	263.97±30.38	185.57±19.03	334.06±42.93	126.5±11.94	245.43±37.32	192.45±26.68	334.42±40.3
Cz	Low	124.49±7.71	252.9±29.44	186.62±18.45	343.37±48.66	123.86±7.65	232.98±27.82	181.47±12.38	327±43.2
	High	121.56±13.37	261.17±39.55	189.53±20.04	354.47±54.06	123.09±7.37	234.04±34.22	183.22±17.11	327.68±41.81
CPz	Low	112.58±21.36	243.43±45.59	187.97±25.89	364.4±66.74	115.9±15.72	230.61±25.99	185.81±23.6	318.58±44.85
	High	109.13±17.5	237.52±37.99	188.07±27.55	368.22±57.75	116.43±15.38	238.58±40.27	189.01±30.55	341.87±61.08
Pz	Low	102.2±24.94	226.52±37.57	174.41±27.66	357.7±63.39	92.4±21.43	237.15±52.44	182.09±32.75	339.89±65.49
	High	97.21±20.41	210.67±27.91	171.49±29.47	362.36±52.72	103.12±31.18	245.17±42.82	183.16±34.64	352.51±59.65

Amplitudes are reported in microvolts (µV) and latencies are reported in milliseconds (ms); means ± SDs provided.

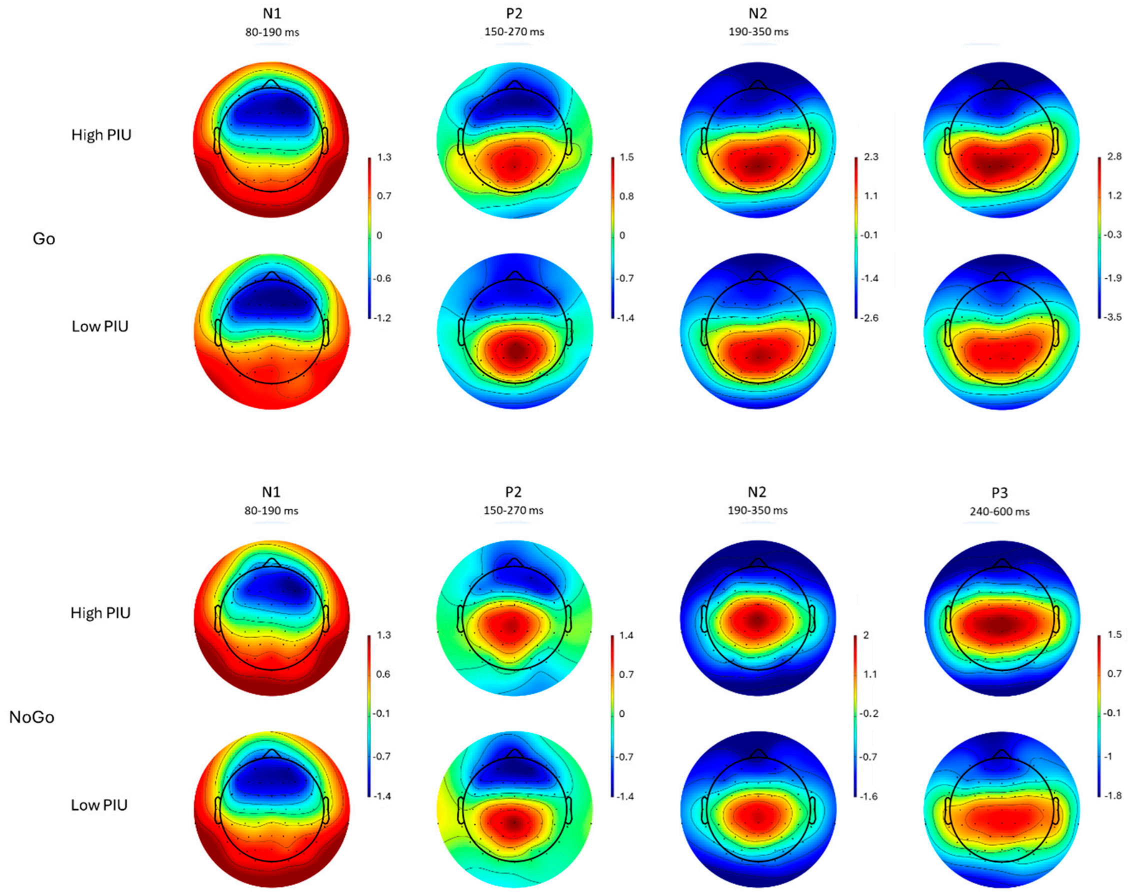

Figure S2. Topographical representation of the N1 (first column), P2 (second column), N2 (third column), and P3 (fourth column) waves in response to Go (upper rows) and NoGo (lower rows) stimuli between high PIU and low PIU groups. No significant differences were observed between groups at any of the electrode sites.
